# Supplementary material for: Plasticity versus stability across the human cortical visual connectome
Source: Nat Commun. 2019 Jul 18;10:3174. doi: 10.1038/s41467-019-11113-z (PMC6639412; doi:10.1038/s41467-019-11113-z)
Supplement: Supplementary file 2 — Reporting Summary [file 41467_2019_11113_MOESM2_ESM.pdf]

nature research

Corresponding author(s): K.V. Haak

Last updated by author(s): 2019/04/05

Reporting Summary

Nature Research wishes to improve the reproducibility of the work that we publish. This form provides structure for consistency and transparency in reporting. For further information on Nature Research policies, see Authors & Referees and the Editorial Policy Checklist.

Statistics

For all statistical analyses, confirm that the following items are present in the figure legend, table legend, main text, or Methods section.

n/a	Confirmed	
☐	☒	The exact sample size (*n*) for each experimental group/condition, given as a discrete number and unit of measurement
☒	☐	A statement on whether measurements were taken from distinct samples or whether the same sample was measured repeatedly
☐	☒	The statistical test(s) used AND whether they are one- or two-sided<br>*Only common tests should be described solely by name; describe more complex techniques in the Methods section.*
☐	☒	A description of all covariates tested
☐	☒	A description of any assumptions or corrections, such as tests of normality and adjustment for multiple comparisons
☐	☒	A full description of the statistical parameters including central tendency (e.g. means) or other basic estimates (e.g. regression coefficient) AND variation (e.g. standard deviation) or associated estimates of uncertainty (e.g. confidence intervals)
☐	☒	For null hypothesis testing, the test statistic (e.g. *F*, *t*, *r*) with confidence intervals, effect sizes, degrees of freedom and *P* value noted<br>*Give P values as exact values whenever suitable.*
☒	☐	For Bayesian analysis, information on the choice of priors and Markov chain Monte Carlo settings
☒	☐	For hierarchical and complex designs, identification of the appropriate level for tests and full reporting of outcomes
☒	☐	Estimates of effect sizes (e.g. Cohen's *d*, Pearson's *r*), indicating how they were calculated

Our web collection on statistics for biologists contains articles on many of the points above.

Software and code

Policy information about availability of computer code

Data collection	Data were collected as part of the WU-Minn Human Connectome Project. All commercial, open source and custom code (and version identifiers) used to collect these data can be found in the associated references and the documentation on https://www.humanconnectome.org/study/hcp-young-adult.
Data analysis	To analyze the data for this study we used: FSL version 5.10; Matlab version R2018a, and Solar Eclipse version 8.1.1.

For manuscripts utilizing custom algorithms or software that are central to the research but not yet described in published literature, software must be made available to editors/reviewers. We strongly encourage code deposition in a community repository (e.g. GitHub). See the Nature Research guidelines for submitting code & software for further information.

Data

Policy information about availability of data

All manuscripts must include a data availability statement. This statement should provide the following information, where applicable:

- Accession codes, unique identifiers, or web links for publicly available datasets
- A list of figures that have associated raw data
- A description of any restrictions on data availability

The source data underlying Figures 1, 2, 3, and 4 are provided as a Source Data file. All relevant MRI data are publicly available at https://db.humanconnectome.org. The heritability analyses require information about the family structure in the HCP data, which is restricted by the HCP due to legal and ethical issues pertaining to confidentiality and privacy of participants. The code for performing these analyses is therefore available from the corresponding author after providing proof of access to HCP restricted data. All other analysis code is readily available from the corresponding author upon reasonable request.

Field-specific reporting

Please select the one below that is the best fit for your research. If you are not sure, read the appropriate sections before making your selection.

☒ Life sciences ☐ Behavioural & social sciences ☐ Ecological, evolutionary & environmental sciences

For a reference copy of the document with all sections, see nature.com/documents/nr-reporting-summary-flat.pdf

Life sciences study design

All studies must disclose on these points even when the disclosure is negative.

Sample size	The sample size (N=380) for this study was determined by the number of available HCP subjects with the following criteria: (1) completion of all 4 rfMRI runs, (2) twin, and (3) twin-status confirmed by genetic testing.
Data exclusions	No additional exclusion criteria were used.
Replication	This study concerns an analysis of heritability of functional connectivity (and cortical thickness and gray matter volume). The functional connectivity estimates were subjected to a test-retest analysis, which confirmed stable results across repeated measures from the same subject sample.
Randomization	N/A (heritability analysis based on existing twin-data)
Blinding	N/A (heritability analysis based on existing twin-data)

Reporting for specific materials, systems and methods

We require information from authors about some types of materials, experimental systems and methods used in many studies. Here, indicate whether each material, system or method listed is relevant to your study. If you are not sure if a list item applies to your research, read the appropriate section before selecting a response.

Materials & experimental systems

n/a	Involved in the study	
☒	☐	Antibodies
☒	☐	Eukaryotic cell lines
☒	☐	Palaeontology
☒	☐	Animals and other organisms
☐	☒	Human research participants
☒	☐	Clinical data

Methods

n/a	Involved in the study	
☒	☐	ChIP-seq
☒	☐	Flow cytometry
☐	☒	MRI-based neuroimaging

Human research participants

Policy information about studies involving human research participants

Population characteristics	This study was performed using publicly available Human Connectome Project (HCP) data. All relevant information can be found in the associated references and in the documents on https://www.humanconnectome.org/study/hcp-young-adult.
Recruitment	This study was performed using publicly available Human Connectome Project (HCP) data. All relevant information can be found in the associated references and in the documents on https://www.humanconnectome.org/study/hcp-young-adult.
Ethics oversight	Washington University in St. Louis Institutional Review Board (IRB).

Note that full information on the approval of the study protocol must also be provided in the manuscript.

Magnetic resonance imaging

Experimental design

Design type	Resting-state and structural MR (twin-study)
Design specifications	4 resting state runs of 1200 time points each (~14.4min per run)
Behavioral performance measures	N/A

Acquisition

Imaging type(s)	Functional, Structural
Field strength	3T
Sequence & imaging parameters	This study was performed using publicly available Human Connectome Project (HCP) data. All relevant information can be found in the associated references and in the documents on https://www.humanconnectome.org/study/hcp-young-adult.
Area of acquisition	Whole brain

Diffusion MRI ☐ Used ☒ Not used

Preprocessing

Preprocessing software	This study was performed using publicly available Human Connectome Project (HCP) data. All relevant information can be found in the associated references and in the documents on https://www.humanconnectome.org/study/hcp-young-adult. Additional spatial smoothing was performed using custom Python scripts.
Normalization	This study was performed using publicly available Human Connectome Project (HCP) data. All relevant information can be found in the associated references and in the documents on https://www.humanconnectome.org/study/hcp-young-adult.
Normalization template	MNI152
Noise and artifact removal	This study was performed using publicly available Human Connectome Project (HCP) data, which was rigorously cleaned according to procedures outlined in the associated references and in the documents on https://www.humanconnectome.org/study/hcp-young-adult.
Volume censoring	None

Statistical modeling & inference

Model type and settings	The heritability analysis involved a variance decomposition of functional connectivity estimates as well as gray-matter and thickness estimates per brain area. Second-level analyses involved linear and quadratic modeling by regression.
Effect(s) tested	The study concerned differences in heritability across brain areas (i.e. as a function of hierarchical level)

Specify type of analysis: ☐ Whole brain ☒ ROI-based ☐ Both

Anatomical location(s): Visual areas determined by a probabilistic atlas

Statistic type for inference (See Eklund et al. 2016)	N/A
Correction	The statistical significance of the functional connectivity estimates was determined using FDR correction.

Models & analysis

n/a	Involved in the study
☐	☒ Functional and/or effective connectivity
☒	☐ Graph analysis
☒	☐ Multivariate modeling or predictive analysis

Functional and/or effective connectivity	Pearson correlation (controlled for the effects of head-motion)
